# Psychometric properties of the Tinnitus Functional Index (TFI): Assessment in a UK research volunteer population

**DOI:** 10.1016/j.heares.2015.09.009

**Published:** 2015-09-28

**Authors:** Kathryn Fackrell, Deborah A. Hall, Johanna G. Barry, Derek J. Hoare

**Affiliations:** a NIHR Nottingham Hearing Biomedical Research Unit, Nottingham, NG1 5DU, UK; b Otology and Hearing Group, Division of Clinical Neuroscience, School of Medicine, University of Nottingham, NG7 2RD, UK; c MRC Institute of Hearing Research, University Park, Nottingham, NG7 2RD, UK; d Nottingham University Hospitals NHS Trust, Nottingham, NG5 1PB, UK

**Keywords:** Outcome instruments, Reproducibility, Reliability, Confirmatory Factor Analysis, Convergent validity, Discriminant validity, Responsiveness

## Abstract

**Objectives:**

Questionnaires are essential for measuring tinnitus severity and intervention-related change but there is no standard instrument used routinely in research settings. Most tinnitus questionnaires are optimised for measuring severity but not change. However, the Tinnitus Functional Index (TFI) claims to be optimised for both. It has not however been fully validated for research purposes. Here we evaluate the relevant psychometric properties of the TFI, specifically the questionnaire factor structure, reproducibility, validity and responsiveness guided by quality criteria for the measurement properties of health-related questionnaires.

**Methods:**

The study involved a retrospective analysis of data collected for 294 members of the general public who participated in a randomised controlled trial of a novel tinnitus device (ClinicalTrials.gov Identifier: NCT01541969). Participants completed up to eight commonly used assessment questionnaires including the TFI, Tinnitus Handicap Inventory (THI), Tinnitus Handicap Questionnaire (THQ), a Visual Analogue Scale of loudness (VAS-Loudness), Percentage Annoyance question, the Beck's Depression Inventory (BDI), Beck's Anxiety Inventory (BAI), and the World Health Organisation Quality of Life-Bref (WHOQOL-BREF). A series of analyses assessed the study objectives. Forty four participants completed the TFI at a second visit (within 7–21 days and before receiving any intervention) providing data for reproducibility assessments.

**Results:**

The 8-factor structure was not fully confirmed for this general (non-clinical) population. Whilst it was acceptable standalone subscale, the ‘auditory’ factor showed poor loading with the higher order factor ‘functional impact of tinnitus’. Reproducibility assessments for the overall TFI indicate high internal consistency (α = 0.80) and extremely high reliability (ICC: 0.91), whilst agreement was borderline acceptable (93%). Construct validity was demonstrated by high correlations between scores on the TFI and THI (*r* = 0.82) and THQ (*r* = 0.82), moderate correlations with VAS-L (*r* = 0.46), PR-A (*r* = 0.58), BDI (*r* = 0.57), BAI (*r* = 0.39) and WHOQOL (*r* = −0.48). Floor effects were observed for more than 50% of the items. A smallest detectable change score of 22.4 is proposed for the TFI global score.

**Conclusion:**

Even though the proposed 8-factor structure was not fully confirmed for this population, the TFI appears to cover multiple symptom domains, and to measure the construct of tinnitus with an excellent reliability in distinguishing between patients. While the TFI may discriminate those whose tinnitus is not a problem, floor effects in many items means it is less appropriate as a measure of change in this subgroup. Further investigation is needed to determine whether these effects are relevant in other populations.

## 1 Introduction

The experience of tinnitus can involve much more than the ‘phantom’ sensation of sound, it can also impact on daily functioning, causing insomnia, difficulties in listening and concentrating, impaired symptom-specific quality of life, and poor psychological well-being (Tyler and Baker, 1983; Robinson et al., 2003; Stevens et al., 2007; Langguth et al., 2011; Nondahl et al., 2011; Pierce et al., 2012). But quantifying the severity of this impact, or how this severity changes as a result of time or intervention, is difficult. Psychoacoustic estimates of tinnitus loudness may partially explain some of the variance attributed to the functional impact or perceived annoyance/intrusiveness of tinnitus (Dauman and Tyler, 1992; Andersson, 2003). But ratings of loudness, annoyance or awareness of tinnitus made using a Visual Analogue Scale (VAS), recommended by some as standalone measures of tinnitus severity, do not correlate strongly with either psychoacoustic or multi-item questionnaire measures of tinnitus (Adamchic et al., 2012). Given that tinnitus is a multi-dimensional symptom, researchers typically rely on multi-attribute self-report questionnaires to quantify tinnitus severity and to assess intervention-related change over time.

Numerous questionnaire measures of tinnitus have been developed to date (for reviews see Fackrell et al., 2014; Meikle et al., 2008; Newman and Sandridge, 2004), and recommended for clinical use (Department of Health, 2009; Langguth et al., 2007; Tunkel et al., 2014). For tinnitus research, the international standards proposed by Landgrebe et al. (2012) calls for the routine use of the Tinnitus Handicap Inventory (THI; Newman et al., 1996), and that researchers define a validated tinnitus questionnaire as at least one of the primary outcome measures. Questionnaires are widely used in tinnitus research to either characterise the participant population (e.g. to aid comparison across different studies; Boyen et al., 2013; Melcher et al., 2013), to measure the effects of experimental intervention (e.g. Hoare et al., 2014a; Song et al., 2013), or to explore correlations between self-reported tinnitus severity and biological observations (e.g. Song et al., 2013; Szczepek et al., 2014). The approaches taken to validate tinnitus questionnaires to date have sometimes limited their utility (Meikle et al., 2008; Fackrell et al., 2014). For example, although the interpretability of the Tinnitus Handicap Questionnaire (THQ; Kuk et al., 1990) has been examined this has not led to defined categories of severity (Newman et al., 1995). The THI was developed specifically as a diagnostic tool with defined categories of severity (Newman et al., 1996; McCombe et al., 2001), and has been criticised for lacking sensitivity to change (Meikle et al., 2007). The Tinnitus Functional Index (TFI; Meikle et al., 2012) was developed to provide (i) comprehensive coverage of the broad range of symptoms associated with tinnitus severity, (ii) reliable measurement of tinnitus severity that distinguishes between individuals from those whose tinnitus is ‘not a problem’ to those whose tinnitus is a ‘very big problem’, and (iii) responsive measurement of change in tinnitus severity. It may therefore have a number of applications in research studies. The questionnaire underwent a systematic process of development to distil an initial item pool of 175 items through two prototypes (prototype 1 had 43 items, prototype 2 had 30 items) to arrive at a final questionnaire containing 25 items each mapping onto one of eight functional subscales (see Meikle et al., 2012 for details). The subscales (factors) were defined through Exploratory Factor Analysis and named as (i) Intrusiveness (items 1–3), (ii) Sense of control (items 4–6), (iii) Cognition (items 7–9), (iv) Sleep (items 10–12), (v) Auditory (items 13–15, (vi) Relaxation (items 16–18), (vii) Quality of life (items 19–22), and (viii) Emotional distress (items 23–25). The development pathway included a process of exploratory factor analysis, assessment of content validity, test-retest reliability, internal consistency, and convergent and discriminant validity. Development of the TFI used data collected from clinics in the USA, primarily specialist tinnitus clinics (42% of participants) and Veterans' Affairs (VA) hospitals (58% of participants). Those recruited from the VA sites tended to be male and experienced a range of co-morbidities, such as Post-Traumatic Stress Disorder (PTSD). Validation of the TFI is understood therefore relative to this mixed clinical population. It cannot be assumed that the questionnaire will show the same properties when administered to a different population. In fact the final 25-item version of the TFI has never been directly subjected to formal psychometric evaluation. The only assessment of validity and reliability was based on analysis of a subset of data collected for the 30-item prototype 2 of the questionnaire, and confirmatory factor analysis was not conducted (Meikle et al., 2012).

Here we examine the properties of the TFI for a general sample of UK adults experiencing tinnitus who presented themselves to take part in a clinical trial guided by quality criteria for the measurement properties of health-related questionnaires (Terwee et al., 2007; see also Fackrell et al., 2014). Specifically, the psychometric validation reported here focuses on assessing (a) the reliability of the 8-factor TFI structure reported by Meikle et al. (2012), i.e. verifying item identification with each factor and the underlying construct using Confirmatory Factor Analysis, and (b) the ability of the TFI to reliably measure tinnitus severity, distinguishing between individual differences in tinnitus-related distress, and responsively measure change in tinnitus severity.

## 2 Materials and methods

### 2.1 Participants and procedure

This was a retrospective analysis of data collected during a two-centre clinical trial conducted at the National Institute for Health Research Nottingham Hearing Biomedical Research Unit (BRU) and the University College London Ear Institute (RESET2, ClinicalTrials.gov ID:NCT01541969; Hoare et al., 2013). For that trial, participants were recruited via adverts placed on the website of the Nottingham Hearing BRU or in local hearing clinics, or to publicity in the national media. Participants reflected a mix of those who had previously attended clinical appointments for their tinnitus, and those who had never sought medical help for their tinnitus. Although none of the participants were receiving any clinical interventions for their tinnitus at the time of assessment, all participants were strongly motivated to seek a specific treatment by volunteering for this clinical trial in which a novel sound therapy for tinnitus was prescribed for a period of 36 weeks of daily use. The intake assessment for eligibility onto the trial provided data for the psychometric validation analysis. Assessment included Percentage Annoyance question, a VAS of tinnitus loudness, the TFI, THI, THQ, the Beck Anxiety Inventory (BAI; Beck and Steer, 1990) and Beck's Depression Inventory (BDI-II; Beck et al., 1996), and the World Health Organisation Quality of Life (WHOQOL-BREF; The WHOQOL group, 1998). In the clinical trial, 391 were assessed for eligibility but 291 were excluded from the trial at either telephone screening or eligibility appointments because they did not meet the inclusion criteria (stated in ClinicalTrials.gov Identifier: NCT01541969, but not relevant for the present study), or withdrew. Hence, 100 participants were allocated to one of the study arms and received treatment. The data contributing to the present study comprised 294 individuals (212 male, 82 female), with an average age of 52.8 years (range: 18 to 82) and tinnitus duration of 9.0 years (range: 4 months to 50 years). We have TFI data at the initial assessment from 285 individuals (two were excluded due of missing data) and of those, 12% reported tinnitus as not a problem (range: 0–17), 27% reported tinnitus as a small problem (range: 18–31), 31% as a moderate problem (range: 32–53), 24% as a big problem and 5% as a very big problem (range: 73–100). This distribution was comparable to that reported by some of the clinical centres participating in the original development of the TFI Protocol 1 (Meikle et al., 2012), with individuals spanning all categories of severity.

Data were collected in accordance with the permissions granted by the Nottingham 1 NHS Research Ethics Committee and the Sponsor (Nottingham University Hospitals NHS Trust) as part of the protocol described in Hoare et al. (2013).

### 2.2 Missing data

Not all participants completed all assessments and only complete questionnaire datasets were analysed. Listwise deletion is considered an effective approach to deal with missing data when only a small amount of data (<5%) is assessed as ‘missing completely at random’ (MCAR) (Schafer and Graham, 2002) and avoids problems associated with over-estimating factors (Tabachnick and Fidell, 2013). This was the case here.

Only those data with fully completed TFI scores on all 25 items were used for analysis of the TFI factor structure, internal consistency and responsiveness (floor and ceiling effects) and so after list-wise deletion the effective sample size was 283. TFI was not completed in 9 cases, and in 2 cases one item was missing (defined as MCAR). Furthermore, analyses of convergent and divergent validity were calculated after list-wise deletion of missing items on the different comparison assessments and so the effective sample size was 247. Forty-seven individuals did not complete all the necessary assessments.

The clinical trial required a second TFI dataset for the 100 enrolled participants, which we used here to assess reproducibility using test-retest reliability and agreement analysis to determine how close repeated measures were to each other. The clinical trial protocol did not specify a required time interval between first and second administration of the TFI, but based on the previous validation (Meikle et al., 2012) and recommendations (Terwee et al., 2007) we conservatively limited reproducibility analyses to data from a subset of 44 participants who completed the TFI twice within an average of 15 days (SD = 7).

### 2.3 Measures

#### 2.3.1 Percentage annoyance

As part of the Tinnitus Case History Questionnaire (TCHQ), participants were asked to state any number between 0 and 100 that represents the percentage of time awake they were annoyed by their tinnitus.

#### 2.3.2 Visual analogue scale of loudness (VAS-Loudness)

As part of the ‘Tinnitus Tester’ computerised test (Roberts et al., 2006, 2008) participants rated the loudness of their tinnitus on a Borg CR100 (VAS) scale (Borg and Borg, 2001). Participants mark the loudness of their tinnitus at any point along the numerical scale, but word descriptors, “extremely weak,” “moderate,” “strong,” “very strong,” and “extremely strong”, are utilised as anchor points which predisposes subjects to interpret it as an ordinal scale. Hoare et al. (2014a) recently reported that test-retest agreement was very high for this element of the Tinnitus Tester.

#### 2.3.3 Tinnitus Functional Index (TFI)

Participants scored each item of the 25 items according to how they felt over the past week. Each item is scored on an 11-point scale, with descriptors at either end of the scale. The procedure for scoring the TFI followed the instructions provided by Meikle et al. (2012). The sum of all scores is divided by 2.5 to give a global score out of 100. Higher scores reflect greater impact on daily functioning. Subscale scores are calculated as the sum of the relevant three or four items.

#### 2.3.4 Tinnitus Handicap Inventory (THI)

The THI measures the effects of tinnitus on everyday function (Newman et al., 1996, 1998; Baguley et al., 2000). Each of the 25 items is rated on a categorical 3-point scale (yes/no/sometimes). The mean global score reflects the sum of all responses with a maximum score of 100 indicating the greatest impact on everyday function. Although subscales of the THI have been proposed (Newman et al., 1996) subsequent analyses have demonstrated that the THI items load predominantly onto a single factor (Baguley and Andersson, 2003; Kennedy et al., 2004) and so for the purposes of analysis here this questionnaire is considered unidimensional.

#### 2.3.5 Tinnitus Handicap Questionnaire (THQ)

The THQ measures overall handicap associated with tinnitus, in particular the effects of tinnitus on hearing and communication, physical health, social and emotional status (Kuk et al., 1990; Robinson et al., 2003). For each of the 27 items, participants indicate their agreement with each item, by assigning a number between 0 (strongly disagree) to 100 (strongly agree). Again, the mean global score reflects the sum of all responses, averaged to give a global score out of 100. Higher scores indicate higher levels of tinnitus handicap. Kuk et al. (1990) recommended a two-factor structure for the THQ, with items relating to factor 1 (physical, emotional and social effects) and factor 2 (hearing and communication ability) considered reliable enough to be used as independent subscales.

#### 2.3.6 Beck's Depression Inventory – II (BDI-II)

The BDI-II provides a measure of depressive symptomatology, in particular mood and physical effects (Beck et al., 1996; Dozois et al., 1998; Segal et al., 2008). Participants select statements characterising how they have felt over the previous two weeks, and each of the 21 items is rated on a categorical scale (0–3 points). Responses are summed to form a global score out of 63, with higher scores indicating higher levels of depressive symptomatology.

#### 2.3.7 Beck's Anxiety Inventory (BAI)

The BAI is a measure of the clinical anxiety (Beck and Steer, 1990; Steer et al., 1993). It lists 21 common symptoms associated with clinical anxiety, such as nervousness and fear of losing control. Participants rate how much they were bothered by each symptom over the previous week on a categorical scale (0–3 points) and, as for the BDI, responses are summed to give a global score out of 63 (higher scores indicate greater anxiety).

#### 2.3.8 World Health Organisation Quality of Life-BREF (WHOQOL-BREF)

The WHOQOL-BREF provides a broad reliable measurement of perceived quality of life embedded in a cultural, social and environmental context (The WHOQOL Group, 1998; Skevington et al., 2004). The WHOQOL-BREF produces four domain scores (physical health, psychological, social relationships and environment) and also includes one facet on overall quality of life and general health (“How would you rate your quality of life?”). This item has 5 response options being (1) “very poor”; (2) “poor”; (3) “neither poor nor good”; (4) “good”; and (5) “very good”. The score is transformed onto a 100 point scale, using the WHOQOL-BREF conversion method (The WHOQOL Group, 1998).

### 2.4 Data screening

Non-normality of data can have adverse effects on the statistics conducted here, in particular the Confirmatory Factor Analysis, so as a first step the TFI data were screened for outliers, linearity and multicollinearity. There was no evidence of univariate outliers in the boxplots and histograms. However Mahalanobis distance statistic indicated that there were nine multivariate outliers with the greatest distance from the rest of the data points (Mahalanobis d-squared: 90.72 to 59.15, p ≤ 0.0001). Kurtosis and skewness did not exceed the recommended cut-off points (for kurtosis = 2.00; skewness = 7.00; Curran et al., 1996). However, Mardia's normalised coefficient estimate was 37, exceeding the recommended value of <5 (Bentler, 2006; Mardia, 1971). This indicates some non-normality in the distribution of the data, requiring control.

The data for all questionnaires (global and subscales scores) met the assumptions relating to multicollinearity and linearity; analysis of tolerance indices and Variance Inflation Factor (VIF) all met the cut-off points of >0.10 and <10, respectively (Menard, 2002; Myers, 2000).

### 2.5 Statistical analysis

#### 2.5.1 Confirmation of the 8-factor structure of the TFI

Confirmatory Factor Analysis was performed in Mplus 7 (Muthén and Muthén, 2012). It was conducted on TFI data to test how the variables observed for our research population fit the 8-factor structure devised by Meikle et al. (2012, Fig. 1). The initial 8-factor model was defined by four properties: (i) The latent constructs: eight first-order factors corresponding to the TFI subscales and one second-order factor corresponding to the global measure “Functional impact of tinnitus”; (ii) Each item (observed variable) loaded only on to its designated first factor without any crossloading (i.e. constrained zero loadings on the other factors); (iii) Residual variance (error/uniqueness terms) associated with each variable (25 items, 8 first-order factors) were assumed to be un-correlated and random (constrained to zero); (iv) The variance of the second order factor was fixed at 1 as it was assumed that the first-order factors are completely explained by the relationship to the second-order factor.

Data were treated as continuous rather than categorical, as the response scale was large (0–10 points) (Mutheén and Mutheén, 2012). To adjust for non-normality in the data and to ensure robust standard errors for parameter estimates and goodness of fit indices, the model was estimated using maximum likelihood parameter estimation adjusted with Satorra–Bentler scaled Chi-square (S–B χ^2^; Satorra and Bentler, 1994; Bentler, 2006; Hu and Bentler, 1999). Caution is needed when interpreting the significance of S–B χ^2^ as it is strongly influenced by sample size and variability in the data (Hu and Bentler, 1998; Brown, 2006).

Factor intercorrelations were performed to indicate the degree to which the factors are related to one another and are potentially overlapping in content. These are examined first before the model included the second-order factor. A degree of overlap is expected between factors such as these as they are purported to be measuring the same underlying construct (functional impact of tinnitus). However, highly correlated factors (>0.85) were taken to indicate that they are not measuring distinct constructs from each other (poor discriminant validity). Weakly correlated factors (<0.30) were taken to indicate that they were highly distinct from each other, and potentially measuring an alternative underlying construct (Brown and Moore, 2012; Brown, 2006).

The criterion for goodness of fit was determined using absolute fit indices S–B χ^2^ (Satorra and Bentler, 1994) and Standardised Root Mean Square Residual (SRMR; Hu and Bentler, 1998; Bentler, 2006) to access the discrepancies between the implied correlations (predicted by the model) and observed covariances. The S–B χ^2^ is assessed relative to the degrees of freedom, and this estimate has a critical ratio cut-off of ≤2.0. Alongside this, a large S–B χ^2^ with p < 0.05 and SRMR that exceeds 0.07 (ideally less than 0.06) were taken to together indicate poor fit and that the model should be rejected. Approximation fit indices were also used. TuckereLewis Index (TLI; Tucker and Lewis, 1973) and Comparative Fit Index (CFI; Bentler, 1990) assessed the model fit to baseline. Values for both should exceed 0.90, and preferably exceed 0.95 (Hu and Bentler, 1999). Root Mean Square Error of Approximation (RMSEA; Steiger and Lind, 1980) measured the discrepancy per degree of freedom. Ideally, RMSEA should be less than 0.05, but values up to 0.08 are considered reasonable when the SRMR value is ≤0.06. RMSEA confidence intervals should also fall within the desired criteria (Brown, 2006; Hu and Bentler, 1999, 1998).

Standardised parameter estimates (β; factor loadings) provided an indication of the magnitude and pattern of the relationship between the latent constructs and the observed variables. Our assumption was that the itemefactor relationship is entirely explained by the influence of the latent construct. Factor loadings exceeding 0.7 are were taken to mean that the majority of the shared variance was explained by the latent construct. Loadings below 0.4 are associated with measurement error or poor explained variance and were taken to indicate a potential source of poor model fit (Brown and Moore, 2012; Floyd and Widaman, 1995).

The Modification Index (MI) and Expected Parameter Change (EPC) were used to identify any misspecification in the parameters of the model. Large modification indices exceeding 3.84 were taken to indicate that if a parameter was freely estimated, rather than fixed or constrained, the overall model fit would significantly improve (Brown and Moore, 2012). The EPC value was used to provide an approximation of the direction or magnitude by the parameter would change in subsequent analysis. Together, they were used to decide, where supported by conceptual foundations, which parameter should be adjusted (Brown and Moore, 2012; MacCallum et al., 1992).

#### 2.5.2 Psychometric properties of the TFI

All statistical analyses were performed in SPSS (v.21.0). Reproducibility, validity and responsiveness of the TFI were assessed.

##### 2.5.2.1 Reproducibility of the TFI

Reproducibility was assessed using three methods; internal consistency, reliability and agreement across testing sessions. *Internal consistency* assesses the extent to which each item in a factor measures the same underlying construct. Cronbach's alpha (α) estimates between 0.7 and 0.9 were taken to indicate acceptable internal consistency (Peterson, 1994; Terwee et al., 2007). *Reliability* compares the degree to which people with tinnitus can be distinguished from each other across two testing sessions, despite measurement error, i.e. the similarity in the variability in scores. Reliability was assessed using Intra-Class Correlations (ICC), with scores >0.70 indicating high reliability (Terwee et al., 2007). *Agreement* relates to the measurement error, and the degree to which each individual's scores collected on two separate time points are in agreement with each other. Agreement was assessed using two methods identifying the limits of agreement (Bland and Altman, 1986) and the Smallest Detectable Change. The limits of agreement method (Bland and Altman, 1986) assumes the mean change score (difference) between repeated measures is zero, and that 95% of mean changes should be within ±1.96 standard deviations of the zero difference score (Bland and Altman, 1986). Limits of agreement were calculated as

$$\text{limits}\;\text{of}\;\text{agreement}\;=\;\bar{d}\text{±}1.96\;\times {\;\text{SD}}_{\text{diff}}$$

where $\bar{d}$ represents the mean difference in scores between the two administrations, the ±1.96 represents two standard deviations, whilst the *SD_diff_* represents the mean difference in standard deviation. This allows for examination of the mean change scores in relation to the change in standard deviation, taking into account the random measurement error. 95% agreement was taken as an indication of high test-retest agreement.

Smallest Detectable Change reflects the extent of expected measurement error and was derived from the Standard Error of Measurement (SEM) between repeated measures Smallest Detectable Change (de Vet et al., 2011; Terwee et al., 2007; de Vet et al., 2006a), where: ${\text{SEM}}_{\text{consistency}}\;\text{=}\;{\text{SD}}_{\text{diff}}\text{/}\sqrt{2}$

$$\text{Smallest}\;\text{Detectable}\;\text{Change}\;\text{=}\;1.96\;\times \;\sqrt{2}\;\times \;\text{SEM}$$

The Smallest Detectable Change score should be comparable to the limits of agreement score to be deemed an acceptable score.

##### 2.5.2.2 Validity of the TFI

Convergent and discriminant validity (the extent to which a questionnaire is measuring the construct it purports to measure; Haynes et al., 1995; Streiner and Norman, 2008) was assessed as Pearson bivariate correlations. To evaluate *convergent validity*, the global TFI scores were compared to THQ and THI global scores in the same population. The TFI was assumed to measure a similar construct and so it was predicted to have high convergent validity with both questionnaires (correlation > 0.60). We predict that the TFI global score will show a weak convergent validity (correlation < 0.6) with VAS-Loudness and Percentage Annoyance, in the same way that THI does (Adamchic et al., 2012).

We expect that general health and quality of life questionnaires measure general constructs of health, not the tinnitus-specific construct measured by the TFI. To evaluate *discriminant validity,* TFI global scores were compared with scores on our general health questionnaires (BAI, BDI-II, WHOQOL-BREF) in the same participants. It was predicted that there would be weak to moderate correlations (<0.6) indicating acceptable discriminant validity.

Secondary analyses on the strength of the relationships between the individual TFI subscales and other questionnaires and their subscales were assessed. Previous evaluations suggest the THI and THQ global scores would correlate with the emotional subscale of the TFI (Kennedy et al., 2004; Baguley et al., 2000; Newman et al., 1996; Kuk et al., 1990). We also predicted that the BDI-II and BAI would moderately correlate with scores on the emotional subscale of the TFI, and that WHOQOL-BREF scores would moderately correlate with the Quality of life subscale of the TFI.

##### 2.5.2.3 Responsiveness of the TFI

With respect to responsiveness, this refers to items that are sensitive to change and confirmation that the questionnaire is able to detect important change (above measurement error; Terwee et al., 2007). Responsiveness was assessed in terms of the number of questions exhibiting floor and/or ceiling effects (having limited capacity for change), and to the value corresponding to the Smallest Detectable Change. Response frequency distributions were examined at item level to detect floor and ceiling effects. Potentially problematic items were predefined as those rated at the lowest or highest possible response option (i.e. 0 or 10 on 10-point scales) by more than 15% of respondents (Terwee et al., 2007). The SEM and Smallest Detectable Change scores were calculated using test-retest data (method described in section 2.5.2.1).

## 3 Results

### 3.1 Inspection of the distribution of scores

Descriptive statistics for all questionnaire measures, including the TFI subscales are shown in Table 1. Scores on tinnitus severity questionnaires were moderate (~40/100 in each case). For depression and anxiety, mean scores were low, although the range was broad. Cumulative frequency distributions for global TFI, THI and THQ are given in Fig. 2. THI global scores were slightly positively skewed towards the lower end of the scales (i.e. 70% of participants scored below 50). THQ global scores had very few higher value scores with all participants scoring less than 70. Compared with these two questionnaires, the TFI global scores appear to be more evenly distributed across the scale, and cover a broad range of scores.

### 3.2 Confirmation of the 8-factor structure of the TFI

**Fig. 1:**
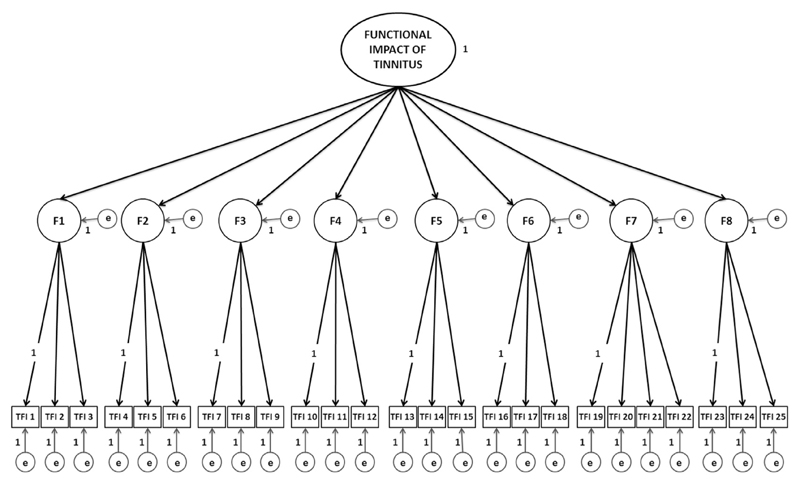
Illustrative diagram of the theoretical 8-factor structure of the TFI assessed by Confirmatory Factor Analysis. The model represents the proposed relationships between the observed variables (items i.e. TFI 1), the first order factors (F1 to F8) and the second-order factor (Functional impact of tinnitus). The model has the following properties: (i) Second-order latent construct: “Functional impact of tinnitus” with the variance fixed at 1. Here, the unidirectional black arrows 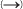 from the second-order factor to the first order factors represent the direct effects of the second-order latent construct onto those factors; (ii) Eight first-order latent constructs: F1: Intrusiveness; F2: Sense of control; F3: Cognition, F4: Sleep; F5: Auditory; F6: Relaxation; F7: Quality of life; F8: Emotional with the variance explained by second-order factor. In this case, the unidirectional black arrows 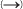 represent the direct effects of the first-order constructs onto the observed measures; (iii) 25 observed variables: TFI item 1 to TFI item 25 with the variance of the first item on each factor fixed at 1, and all items have zero loadings on the other factors; (iv) The unidirectional grey arrows 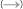 represent the residual variance (e) associated with each variable (25 items; 8 first-order factors), which were constrained to zero. F1 = Intrusiveness; F2 = Sense of control; F3 = Cognition, F4 = Sleep; F5 = Auditory; F6 = Relaxation; F7 = Quality of life; F8 = Emotional; e = residual variance (error and uniqueness terms).

**Fig. 2:**
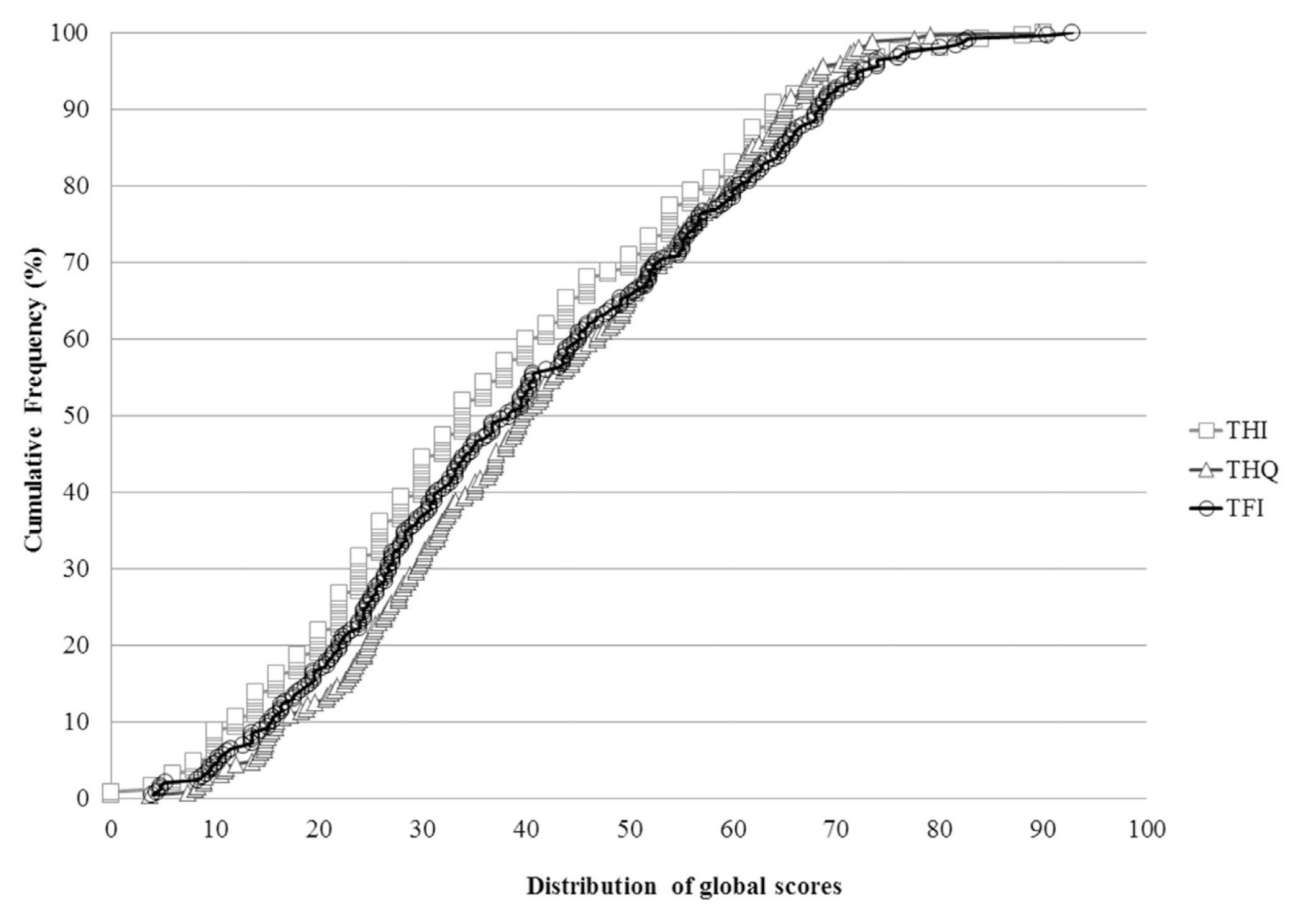
Cumulative frequency distributions of Tinnitus Functional Index (TFI), Tinnitus Handicap Inventory (THI), and Tinnitus Handicap Questionnaire (THQ) global scores. The percentage of responses for 247 participants on the three different tinnitus questionnaires completed. The graph indicates that the TFI global scores are evenly distributed across the scale, i.e. 100% of participants scored below 90, whilst the THI and THQ global scores distributed towards the lower end, i.e. 70% of participants scored below 50 on the THI and all participants scored less than 70 on the THQ.

The initial 8-factor model shown in Fig. 1 was subjected to Confirmatory Factor Analysis.

#### 3.2.1 Factor intercorrelations

Correlation between the first-order factors ranged from very weak (*r* = 0.11) to extremely strong (*r* = 0.85), but most were strong, with 85% above 0.60 (Table 2). The Auditory factor showed unacceptably weak correlations with all the other factors, from an extremely weak correlation with Sleep (*r* = 0.11) to a moderate correlation with Quality of life (*r* = 0.43).

#### 3.2.2 Original model fit

S–B χ^2^ was large and significant (χ^2^: 578.95; p < 0.001) suggesting poor model fit. However, the S–B χ^2^ relative to the degrees of freedom (df = 267) was only marginally higher (2.1) than the critical ratio cut-off (≤2.0), suggesting the fit could improve with modifications (Schreiber et al., 2006). The SRMR indicated an acceptable fit. Approximation fit indices also suggested that the model was acceptable albeit less than optimal (Table 3). The TLI and CFI scores were both acceptable, whilst the RMSEA score indicated reasonable fit. Consequently, at this stage, factor loading estimates and modification indices were examined to identify the potential source of the “less than optimal” model fit. The identified parameters were re-specified accordingly, if they improved the model fit and if they were conceptually justified.

#### 3.2.3 Factor loading estimates

The standardised and unstandardised parameter estimates, R-square values and the standard errors are summarised in Table 4. Standardised parameter estimates for the model revealed high factor loading estimates (>0.70) for all the items with their designated factor, except for items 1 and 4, which had factor loadings of 0.68 and 0.57, respectively.

The Auditory and Sleep factors had the weakest factor loadings with the second-order factor. The Auditory factor (F5 in Table 4) loading estimate was only 0.31 indicating a very weak relationship to the second-order factor. The squared factor loadings mirrored these findings (see R^2^ in Table 4). For instance, the Sense of control factor only accounted for 33% of the variance in Item 4. The second-order factor of only accounted for 39% of the variance in the Sleep factor (F4 in Table 4) and of most concern, only 9% of the variance in the Auditory factor. The rest of the squared factor loadings for the factors and items ranged from 0.45 to 0.95. From this we conclude that the Auditory factor makes considerably less contribution to the global ‘Functional impact of tinnitus’ construct than do the other seven factors.

#### 3.2.4 Modification index (MI) and expected parameter change (EPC)

Findings indicated the presence of three large MIs that were constrained in the initial 8-factor model. Error covariance (uniqueness) was identified between item 16 “*How much has your tinnitus interfered with your quiet resting activities?*” and item 18 “*How much has your tinnitus interfered with your ability to enjoy* ‘*peace and quiet*’*?*” (MI: 35.62; EPC: 1.45) on the relaxation subscale, and between item 19 “*How much has your tinnitus interfered with your enjoyment of social activities?*” and item 21 “*How much has your tinnitus interfered with your relationships with family, friends and other people?*” (MI: 25.72; EPC: 1.05) on the Quality of life subscale. Inspection of these items indicated that the large error variance might be attributable to the similarity of the question wording. Therefore, these were freely estimated in the re-specified model (Table 4).

Cross-loading was identified for item 22 (MI: 25.93; EPC: 1.22). Even though item 22 strongly loaded (0.70) onto the Quality of life factor in the initial model; results indicated that it also loaded onto the Cognitive factor. Item 22 asks “*How often did your tinnitus cause you to have dif*fi*culty performing your work or other tasks, such as home maintenance, school work, or caring for children or others?*”. In this context, the focus is on assessing “difficulties in performing work or tasks” which could be attributed to cognitive processes. There is logic to this cross-loading and although this might marginally lower the loading estimates these parameters were freely estimated in the respecified model.

#### 3.2.5 Model fit for re-specified model

**Fig. 3:**
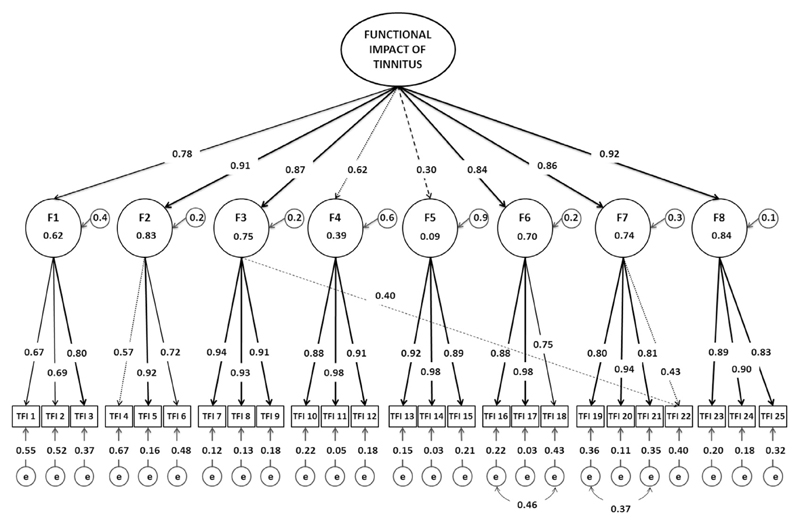
Illustrative diagram of the re-specified 8-factor model including standardised parameter estimates and r-squared values. The diagram represents the re-specified model results. The standardised parameter estimates indicate the strength of the association between the observed variables, first-order factors and the second-order factor. The unidirectional arrows represent the direct effects of the latent constructs. The solid black unidirectional arrow 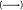 indicates a very strong association (>0.70). The dotted unidirectional arrows 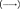 indicate moderate associations with loading values below 0.65. The dash line unidirectional arrows 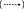 indicate poor associations below the recommended cut-off (<0.40). The residual variance (e) represents the error and unique variance associated with each of the items and the factors. The bidirectional curved arrows 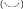 represent the association between the error variance. The dotted unidirectional arrow 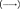 from first-order factors; Sense of control (F3) and Quality of life (F7) to the observed variable TFI22 indicates the cross-loading for item 22. F1 = Intrusiveness; F2 = Sense of control; F3 = Cognition, F4 = Sleep; F5 = Auditory; F6 = Relaxation; F7 = Quality of life; F8 = Emotional; e = residual variance (error and uniqueness terms).

The SRMR improved and the approximation fit indices were all within desirable limits (Table 3), although S–B χ^2^ remained <0.001, the χ^2^/df ratio was now 1.89 so within the critical cut-off of <2.0. RMSEA improved slightly (to 0.056), while TLI and CFI were similar to those of the original model (Table 3). Re-specification of the parameters identified as error covariance marginally reduced the factor loading estimate for those items associated with the error, suggesting that the items loading estimates were previously inflated with unique variance. Although factor loading estimates were expected to marginally fall due to the cross-loading, re-specification of the parameters to adjust for cross-loading item 22 substantially reduced the loading estimates for this item on both factors (to 0.4 and 0.43, Table 4). This was unexpected. The standardised parameter estimates and R-square values for the final model are given in Fig. 3.

### 3.3 Psychometric properties of the TFI

#### 3.3.1 Reproducibility of the TFI

Inter-item correlations ranged 0.055 to 0.904 (Appendix A). Most notably, the Auditory subscale items 14 and 15 exhibited extremely low correlations (*r* ~ 0.1) with the Sleep subscale items 10, 11 and 12. Otherwise items generally showed low to moderate correlations with one another, indicating expected variability in item content. Alpha estimates for the global TFI scores were high (α = 0.80, Table 1). Alpha estimates for the TFI subscales were also extremely high, except for the Intrusiveness subscale which was low (0.58), and considerably lower than that reported by Meikle for prototype 2 where α = 0.85. This lower alpha estimate further indicates poor fitting items within this dataset.

Table 5 summarises test-retest reliability and agreement between two repeated measures. ICC for the TFI global score was 0.91, indicating excellent reliability, and all subscale scores showed similarly high reliability with ICCs ranging 0.81 to 0.95.

In terms of agreement, the Smallest Detectable Change and limits of agreement values for the global and each of the subscale scores were largely comparable. For example, the TFI global scores had a Smallest Detectable Change score of 22.4, whereas the limits of agreement score was 22.2. The Smallest Detectable Change scores are all slightly different than the limits of agreement scores because the SEM_*consistency*_ score (i.e. SEM_*consistency*_ of 8.1) is considered in the calculation of the Smallest Detectable Change, but not in the calculation of the limits of agreement.

**Fig. 4:**
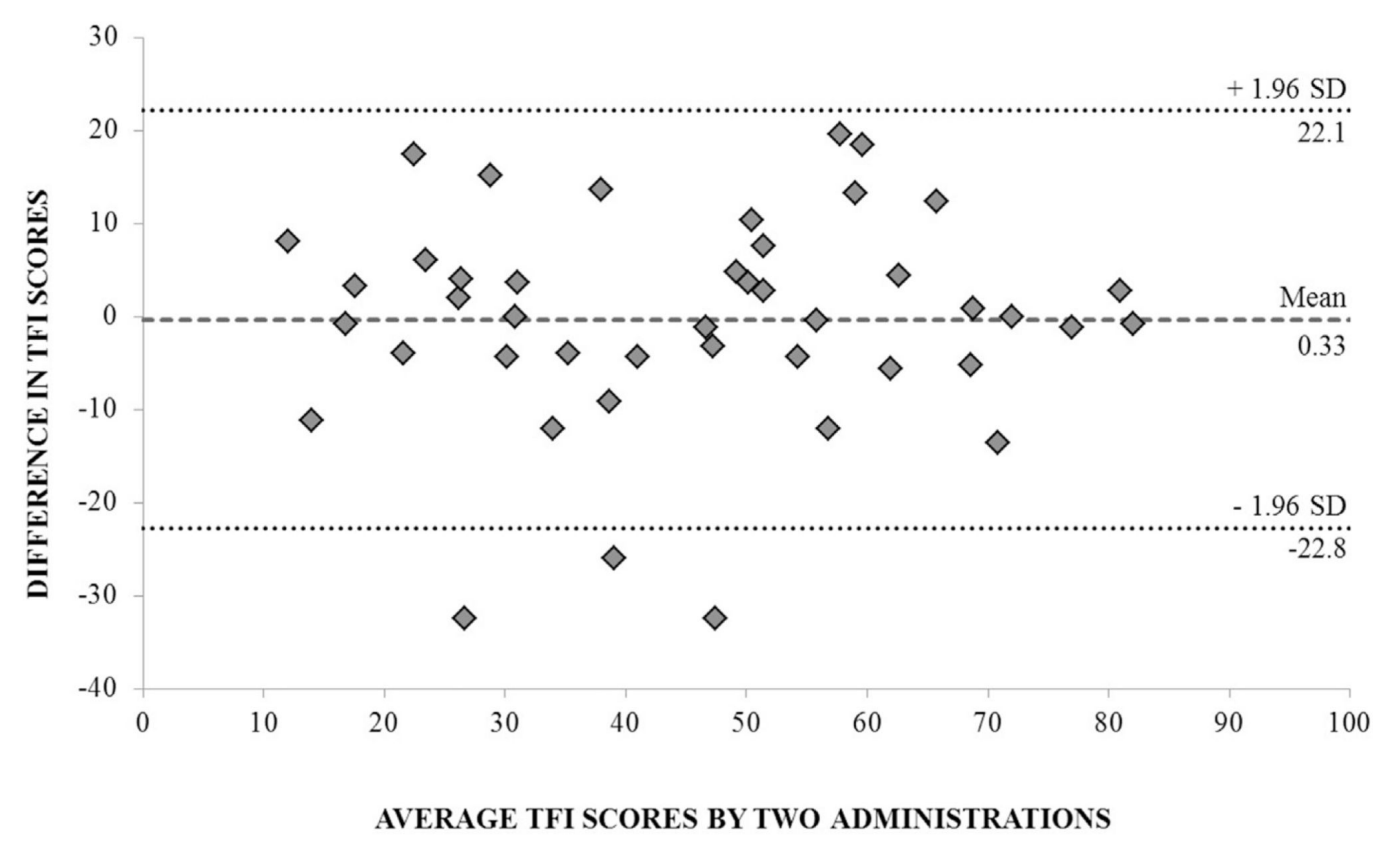
Blande–Altman plot of test-retest agreement for repeated measures of the TFI global scores. The limits of agreement are represented as ±2 standard deviations from the standard error of measurement. The dotted line denotes the 95% limits of agreement for the TFI global scores. 93% of scores are within the limits of agreement, suggesting marginal measurement error between the repeated measures. Dashed line = mean difference. Dotted lines = limits of agreement (1.96 × SD of the mean difference).

Some of the repeated measure change scores in TFI global and subscale scores were not within the identified agreement limits. For three participants, the differences between the TFI global scores were outside the defined limits of agreement (more than 22.2 points below the mean difference; Fig. 4). 95% agreement between scores was observed for only one of the eight TFI subscales, Sense of Control, but not the global score (Table 5).

#### 3.3.2 Validity of the TFI

Pearson's correlation coefficients between the global scores on all measures (TFI, THI, THQ, VAS-Loudness, Percentage Annoyance, BDI-II, BAI and global WHOQOL-BREF) are displayed in Table 6.

For convergent validity, results were as predicted. TFI global scores showed strong positive correlations with the THI and THQ global scores (*r* = 0.82 in both cases) and moderate positive correlations with the VAS-Loudness (*r* = 0.46) and Percentage Annoyance (*r* = 0.58). Therefore, the TFI demonstrates acceptable convergent validity indicating that it measures a tinnitus construct that is similar to that measured by other multi-item tinnitus questionnaires.

For most of the TFI subscales, moderate to strong positive pairwise correlations were observed with THI and the THQ global scores (see values for *r* reported in Table 7). However, when the influence of the remaining subscales were held constant, partial correlation coefficients demonstrated that only the Emotional subscale remained meaningful with a moderate to weak correlation (THI, *pr* = 0.31 and THQ, *pr* = 0.29, respectively) and the Auditory subscale with a moderate correlation (THQ *pr* = 0.41). To confirm the strength of the association between the TFI subscales and the THI and THQ global scores, a series of multiple linear regression analyses were also conducted (see estimated values for β reported in Table 7). These beta values (β) mirrored the same pattern as shown by the partial correlations indicating that the TFI is measuring similar properties of emotional distress as in the THI and THQ and of auditory difficulties as in the THQ.

Finally, correlations between TFI subscales and the two major subscales of the THQ were examined (Table 8). The THQ subscale 1 assesses the physical, emotional and social effects of tinnitus, while the THQ subscale 2 assesses hearing and communication ability. THQ subscale 1 scores correlated strongly with most TFI subscales, while THQ subscale 2 scores correlated moderately or strongly with all TFI subscales. However, when the influence of remaining subscales were held constant, partial correlation coefficients demonstrated that only the TFI Auditory subscale remained meaningfully associated with THQ subscale 2, with a strong correlation (*pr* = 0.71). TFI Emotional and Sleep subscales remained meaningfully associated with THQ subscale 1, with a moderate correlation (*pr* = 0.36 and *pr* = 0.31 respectively). Acceptable convergent validity was therefore only shown by the TFI Auditory subscale and the THQ hearing and communication subscale.

For discriminant validity, results were also as predicted. TFI global scores correlated moderately with BDI-II (r = 0.57), BAI (r = 0.39), and WHOQOL-BREF global item scores (r = 0.48). Therefore, the TFI demonstrates acceptable discriminant validity and is concluded to measures construct(s) that are distinct from those measured by more general health domains.

Partial correlations between individual TFI subscales and general health, with the remaining subscales held constant, yielded a distinct pattern of results. As predicted, the TFI Emotional subscale correlated significantly with all three general health questionnaires (Table 7). Against our prediction, the Quality of life subscale showed only a *weak* negative correlation with WHOQOL-BREF (*pr* = −0.13). The only other notable correlation was the weak correlation between the BDI-II and the TFI Cognitive subscale (*pr* = 0.25). Beta values (β) estimated as part of a series of multiple linear regression mirrored findings from the partial correlation analyses, although they were marginally higher. The Emotional subscale again had the highest β, showing moderate associations with the BDI-II, BAI and WHOQOL-BREF (Table 7). The Cognitive subscale showed a moderate association with the BDI-II, perhaps indicating some sensitivity to aspects of cognitive difficulty associated with generalised depression. Overall, these results suggest an acceptable degree of discriminant validity. The partial correlations and beta values indicate as expected that the BDI-II and BAI are greatly associated with the emotional subscale, whilst unexpectedly the WHOQOL-BREF only showed a small association with the Quality of life subscale.

#### 3.3.3 Responsiveness of the TFI

**Fig. 5:**
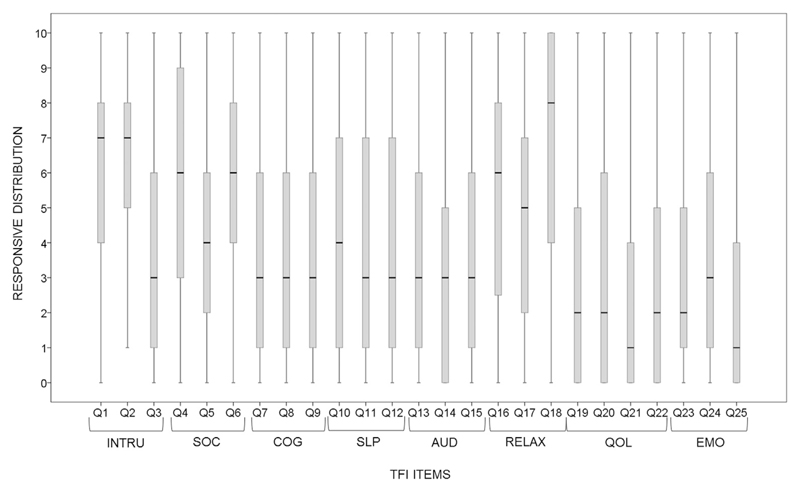
Response frequency distributions for each Tinnitus Functional Index item within their subscales allowing for examination of floor and ceiling effects. Ceiling effects are evident from the position of the upper quartile and medium on the upper end of the scale, i.e. on response options 9 and 10. Item 4 and item 18 both show ceiling effects. For example, the upper quartile for item 18 is at the end of the scale, indicating that 25% of people endorsed the highest category (10) and the medium indicates that over 50% of participants selected the response options 8, 9, and 10. The floor effects are evident by the position of the first quartile and medium on the lower end of the scale, i.e. on response options 0 and 1. Fifteen items showed floor effects. For example, the lower quartile and medium for item 25 indicates that 50% of participants selected response options 1 and 0. This suggests that these items are limited in their detection of change in tinnitus severity, reducing the responsiveness of the TFI. TFI = Tinnitus Functional Index; INTRU = Intrusiveness; SOC = Sense of control; COG = Cognition; SLP = Sleep; AUD = Auditory; REL = Relaxation; QOL = Quality of life; EMO = Emotional.

Response frequency distributions for each item on the TFI were examined for floor and ceiling effects (Fig. 5: Appendix B). Seventeen out of 25 items failed to meet the *a priori* definition of non-significant floor or ceiling effects (i.e. ratings of either 0 points (floor effect) or 10 point (ceiling effect) being observed in no more than 15% of respondents on the 11-point scale). More specifically 15 items showed floor effects, with ‘0’ being observed for between 16 and 41% of participants (items 24, 13, 10, 9, 8, 11, 12, 15, 23, 14, 20, 19, 22, 21, and 25, respectively). Two items showed a ceiling effect, with responses of 10 being observed for 22% and 25% of the population (items 4 and 18, respectively).

Smallest Detectable Change scores were identified for the TFI global and subscale scores (Table 5). For the TFI global score, the Smallest Detectable Change score was above or below 22.4. Change scores above 22.4 were taken to detect true changes related to worsening or improvement of tinnitus. For example, if a change in TFI global score of 23 was observed, it is reasonable to assume that this reflects real change rather than measurement error. For the TFI subscales, Smallest Detectable Change scores were in general larger than the global score Smallest Detectable Change, ranging from 21.1 (Intrusiveness subscale) to 38.5 (Relaxation subscale). Therefore, the subscale scores would have to have large changes before a “true change” is represented.

## 4 Discussion

Although only recently developed, the TFI has been implemented as a baseline assessment and outcome measure in numerous research studies (including Henry et al., 2015; Krings et al., 2015; Michiels et al., 2014; Shekhawat et al., 2014; Wilson et al., 2015). The psychometric evaluation performed here however provides the first account of how reliably the TFI measures tinnitus severity and how well it distinguishes between individual differences in tinnitus-related distress in a research population. We raise a number of important points for discussion and reach a number of specific conclusions on the use of the TFI in a UK research population:

### 4.1 The global TFI is a composite measure of the functional impact of tinnitus

According to our psychometric evaluation, the TFI generally performed adequately as a good measure of functional impact of tinnitus. It has good construct validity and converged on the same construct of tinnitus severity as other multi-item tinnitus questionnaires. In particular, the emotional aspects as measured by the TFI were strongly associated with the global THI and THQ. From the discriminant validity findings, the TFI score is clearly a different measure from those of generalised depression, anxiety, or quality of life.

Confirmatory Factor Analysis broadly confirmed consistency with the eight-factor structure proposed by Meikle et al. (2012). However, there was some evidence of poor fit to the initial model and this improved when the questionnaire was re-specified to account for error covariance between two pairs of items and cross loading of one item onto two factors. Hence, an alternative TFI structure that slightly differed from that proposed by Meikle et al. (2012) was required to best explain the data captured in the general tinnitus population. The next section discusses several other properties in which discrepancies with the original TFI validation were observed, or new concerns are raised.

### 4.2 The TFI auditory subscale does not reliably contribute to the functional impact of tinnitus

Inspection of the first-order factors (corresponding to the subscales) revealed a problem with the Auditory factor in so far as it appeared to be unrelated to the other factors and in turn the underlying global construct of the functional impact of tinnitus. Hence, scores on the auditory subscale provide little additional information about the functional impact of tinnitus and in fact are likely to undermine the global TFI score. Internal consistency and reliability of the Auditory factor were both high, indicating that the items measure the same underlying construct, and that the factor can differentiate between individuals. It would therefore be reasonable to consider the auditory subscale as a stand-alone measurement tool. In our research population, the TFI therefore seems to be measuring two distinct theoretical constructs (a composite measure of the functional impact of tinnitus and a specific auditory domain).

Despite the different tinnitus populations, our finding is consistent with the analyses of Meikle et al. (2012) who also observed weak intercorrelations between the Auditory factor and the other seven factors. The authors suggested that there is perhaps, either “a general tinnitus severity factor underlying all eight subscales…[or] a general tinnitus severity factor underlying seven of the eight subscales, with the Auditory subscale representing an underlying specific factor” (p.20). A general issue may be the difficulty patients sometimes have in determining their tinnitus problems as distinct from the problems they have because of hearing loss (Ratnayake et al., 2009).

### 4.3 There is mixed evidence that the TFI Intrusiveness subscale is a reliable unitary construct and the items that tend to be used most as single-item visual analogue scales are poorly associated with the global construct (functional impact of tinnitus)

Our findings indicate that the Intrusiveness subscale had unacceptably low internal consistency indicating that the three items (TFI 1–3) do not measure the same underlying construct, but instead may be distinct from each other. Questions relate to percentage of time that the respondent is consciously aware or annoyed by the tinnitus (TFI 1 and 3, respectively), and a rating of how strong or loud is the tinnitus (TFI 2). There is no further evidence of this discrepancy in the inter-item correlations or the CFA; all the items had acceptably high loading values.

Some researchers use variants of these questions as singleitem visual analogue scales to assess tinnitus severity and to measure treatment-related change (TFI 2 and 3 are good examples). Correlations between global TFI score and the VAS-Loudness and Percentage Annoyance were moderate at best. From this, we conclude that single item measures are not sufficient to capture the complexity of tinnitus symptomatology captured by multi-item instruments. The limitations with single items are widely recognised, they are variably reported to be psychometrically weak, with poor validity, low reliability and poor responsiveness (Adamchic et al., 2012; Hobart et al., 2007; Goebel and Hiller, 1994; Nunnally, 1967) yet are sometimes used as diagnostic or outcome measures in research (e.g. Tass et al., 2012; Vanneste et al., 2013). We recommend single-item measures are not used to measure the therapeutic effectiveness of interventions.

### 4.4 The TFI quality of life subscale does not assess the full multi-attribute nature of quality of life

Here we observed that the TFI Quality of life subscale did not converge with the single item facet on overall quality of life and general health. It is therefore unlikely that the TFI Quality of life subscale is a surrogate marker for the generic construct of Quality of Life used in health research. Health-related QoL is a ubiquitous concept that has different philosophical, political and health-related definitions, but the World Health Organization (1997) describe it as “individuals' perceptions of their position in life in the context of the culture and value systems in which they live and in relation to their goals, expectations, standards and concerns”. Correspondingly, the WHOQOL-BREF measures four domains associated with quality of life; physical health, psychological health, social relationships, and environment. To avoid the risk of making a Type 1 error by making multiple comparisons between the TFI and these different domains, we evaluated only the single item. However, these findings enable us to draw the preliminary conclusion that health-related QoL is unlikely to be captured by the items in the TFI Quality of life subscale. This is explicable given the development of the TFI which collapsed only ‘Social Distress’, ‘Leisure’, and ‘Work’ domains to create the Quality of life subscale (Meikle et al., 2012), certainly leaving out physical health.

### 4.5 The global TFI score may be poorly responsive to treatment-related change in a research population

Arguably, the single most important factor for clinical trials is the assessment of outcome. Primary outcomes provide the means to determine what interventions are effective and hence to influence therapeutic management strategies. It is essential to identify a primary outcome tool that measures symptom categories and changes that are expected to occur according to the aims of the treatment under investigation (Landgrebe et al., 2012; Langguth et al., 2007).

Substantial floor effects on many items indicated that the TFI would be somewhat limited in its responsiveness to detecting treatment-related benefits in this study population. From our sample of research participants, scores on the majority of the items were close to floor, particularly for items in the Cognitive, Sleep, Auditory and Quality of life subscales. This could be an indication that the items are not related to the underlying construct or that the wording of the items may be misleading indicating a “no problem” response (Terwee et al., 2009; Streiner and Norman, 2008). However, the latter is not indicated by any of the other findings from this study. Further research is warranted to replicate our findings and if necessary to reassess the items for inclusion or their wording. It may be that the TFI is suboptimal for use as a tinnitus outcome instrument in a research volunteer population.

Statistically significant differences in treatment effects provide information only on the error rate between the two interventions. Identification of a minimal change that is clinically meaningful is fundamental in health research and clinical trials. Following Jaeschke et al. (1989), our operational definition of a minimal clinically important difference is the smallest difference in score in the domain of interest which *patients* perceive as beneficial. Generally, a minimal clinically important difference involves patient perception. An important step towards determining minimal important differences is to evaluate the smallest change above measurement error, i.e. the Smallest Detectable Change (Landgrebe et al., 2012; Terwee et al., 2009; Revicki et al., 2008; de Vet et al., 2006a; de Vet et al., 2006b).

Test-retest data was used to identify a Smallest Detectable Change score and results indicated that a change in the TFI global score of at least 22.4 points would be required to represent a true change above measurement error. The magnitude of this change is considerably larger than the 13-point difference proposed by Meikle et al. (2012) as a clinically meaningful change. This discrepancy was larger than expected. It is possible that the statistical method used by Meikle et al. (2012) provided a too conservative estimate. Meikle et al. (2012) used an anchor-based approach and Lipsey's criterion group approach (Lipsey, 1983, 1990), using grouped responses from a global question on self-reported change to anchor the changes on the TFI. Such anchor-based methods do not account for measurement precision which could potentially result in unrealistically low cutoffs that sit within the measurement error (de Vet et al., 2006b; Crosby et al., 2004). Consequently, a change score of 13 points might not be a realistic reflection of true change in score and may still include measurement error.

Given the potential for conflicting results simply arising from whether anchor-based or distribution-based methods are used to calculate the clinically meaningful change score, we recommend an integrated approach using both to identify a clinically meaningful change score that is comparable across methods (Crosby et al., 2004).

## 5 Conclusions and recommendations

This study provides an overview of the psychometric properties of the TFI when used in research. Our findings lead us to draw the following conclusions:

### 5.1 Not all of the TFI subscales contribute equally to the composite measure of the functional impact of tinnitus. In particular, the auditory subscale score does not contribute to the functional impact of tinnitus

Generally speaking, the TFI provides an adequate composite measurement tool for evaluating the functional impact of tinnitus. However, researchers should remain aware that not all of the TFI subscales contributed equally to the global TFI scores measured in this tinnitus population. In particular, the Auditory subscale appeared to be measuring something different from that of the other subscales. Further improvements in the TFI that tailors this measurement tool are warranted. We note that Meikle et al. (2012) also observed a similar pattern in their clinical population. One priority area for future research would therefore be to explore the impact of removing the auditory subscale. For example, the Auditory subscale score could be calculated and reported separately.

### 5.2 The TFI quality of life subscale does not assess generic quality of life

Our current recommendation is to include a multi-attribute health-related QoL measure in research that asks questions about quality of life, and not to rely on this particular TFI subscale for a meaningful interpretation of generic quality of life. Future studies should consider the inclusion of a well-established quality of life scale that generates a global score which seems at least to be responsive to treatment-related change in a clinical population of patients with tinnitus. The HUI3 would seem to be a good candidate (Maes et al., 2011).

### 5.3 The global TFI score and subscale scores may be poorly responsive to treatment-related change in a research population

We provide a cautious recommendation that the TFI is suboptimal for use as a tinnitus outcome instrument in a research volunteer population. However, this warrants further independent replication. Poor responsiveness could be mitigated to some degree by specifying a lower cut-off score as a participant inclusion criterion, one that is at least as large (if not greater) than the Smallest Detectable Change score. As for making a recommendation about the Smallest Detectable Change score that is clinically meaningful and which considers measurement precision, our recommendation is to use the Smallest Detectable Change score of 23 until further research suggests otherwise.

Psychometric validation is an ongoing process that requires continuous evaluations in a variety of populations to provide the much needed evidence that the measurement tool is appropriate and performs as anticipated (Noble, 1998). For the TFI, the various evaluations are ongoing internationally and so we look forward to better understanding and optimising the use of this questionnaire for research and clinical practice alike.

## Appendix

Appendix

## Figures and Tables

**Table 1:** Descriptive statistics and internal consistency. The maximum score is 100, except for BDI and BAI where the maximum score is 63. Values presented in bold indicate poor internal consistency below the recommended criteria (α < 0.7).

Questionnaire/subscale	# Items	Descriptive statistics			Internal consistency	Sample size
		Mean	SD	Range	α	N
Tinnitus Functional Index (TFI)^a^	25	40.6	20.1	4–93	0.80	283
*Intrusiveness*	3	52.8	21.1	6–93	**0.58**
*Sense of control*	3	53.9	23.2	0–100	0.75	
*Cognition*	3	35.8	27.1	0–100	0.95	
*Sleep*	3	39.6	32.3	0–100	0.94	
*Auditory*	3	34.0	27.3	0–100	0.95	
*Relaxation*	3	54.6	29.2	0–100	0.93	
*Quality of life*	4	28.2	25.4	0–100	0.90	
*Emotional*	3	30.3	26.3	0–100	0.91	
Tinnitus Handicap Inventory (THI)^b^	25	37.6	20.1	0–90	0.91	247
Tinnitus Handicap Questionnaire (THQ)^c^	27	41.3	17.9	3–90	0.91	247
*Social, emotional and physical functioning*	15	39.4	23.2	1–91	0.94	
*Hearing ability and unease*	8	40.4	22.7	0–98	0.86	
Beck's Depression Inventory-II (BDI-II)^d^	21	8.4	8.2	0–51	0.92	247
Beck's Anxiety Inventory (BAI)^e^	21	5.0	6.4	0–44	0.90	
WHOQOL-BREF *global item 1*^f^	1	39.1	8.0	10–50	–	
Tinnitus loudness VAS-L	–	50.1	22.0	1–100	–	
Tinnitus annoyance rating	–	39.8	30.4	1–100	–	

SD = standard deviation; α = Cronbach's alpha estimates.

a (Meikle et al., 2012).

b (Newman et al., 1996).

c (Kuk et al., 1990).

d (Beck et al., 1996).

e (Beck and Steer, 1990).

f (WHOQOL group, 1998).

**Table 2:** Correlations between first-order factors in the Confirmatory Factor Analysis. The correlations between the first-order factors were in general strong, with 85% above 0.60. The Auditory factor showed the weakest correlations with all the other factors. 1 = Intrusiveness; 2 = Sense of control; 3 = Cognition; 4 = Sleep; 5 = Auditory; 6 = Relaxation; 7 = Quality of life; 8 = Emotional. Values presented in bold are below or above the recommended criteria (<0.30 to >0.85).

Factor	1	2	3	4	5	6	7	8
(1) Intrusiveness	1							
(2) Sense of control	0.842	1						
(3) Cognitive	0.640	0.795	1					
(4) Sleep	0.507	0.570	0.562	1				
(5) Auditory	0.328	**0.223**	0.330	**0.114**	1			
(6) Relaxation	0.655	0.814	0.725	0.613	**0.239**	1		
(7) Quality of life	0.655	0.733	0.782	0.465	0.413	0.687	1	
(8) Emotional	0.676	**0.855**	0.784	0.543	**0.197**	0.722	**0.855**	1

**Table 3:** Summary of the model fit. Model based on proposed factor structure and re-specified model for final factor structure with modifications. Following modifications, model fit improved with all fit statistics, but the S–B χ^2^, within the desired limits. Therefore the re-specified model represents the best fit of this population data. S–B χ^2^ = Satorra & Bentler adjusted Chi-square; SRMR = Standardised Root Mean Square Residual; TLI = TuckereLewis Index; CFI = Comparative Fit Index; RMSEA = Root Mean Square Error of Approximation.

Models	Modifications	S–B χ^2^ (*df*)	χ^2^/*df*	p-value	TLI	CFI	SRMR	RMSEA (95% CI)
Original model	None	578.947 (267)	2.17	<0.001	0.939	0.946	0.06	0.064 (0.057–0.071)
Re-specified model	Error covariance, cross-loading (Q22 with F3)	498.484 (264)	1.89	<0.001	0.954	0.959	0.056	0.056 (0.048–0.064)

**Table 4:** Parameter estimates, R-squared values and Standard Error for the proposed Confirmatory Factor Analysis Model and Re-specified Model. The factor loadings (standardised/unstandardized), standard errors and squared factor loadings (R-squared) for all 25 observed variables (Items) and the eight first-order factor (factor loadings). Two loading estimates representing the cross-loading for Item 22 are given for the re-specified model. The values presented in bold have poor associations with their designated factor, all below the recommended cut-off <0.40. β = Standardised parameter estimate; B = Unstandardised parameter estimate; SE = Standard Error; R_2_ = R-squared. TFI = Tinnitus functional Index; F1 = Intrusiveness; F2 = Sense of control; F3 = Cognition, F4 = Sleep; F5 = Auditory; F6 = Relaxation; F7 = Quality of life; F8 = Emotional.

First order factor	Observed variable	Original model				Re-specified model			
		β	B	SE	R^2^	β	B	SE	R^2^
Intrusiveness	TFI 1	0.68	1.00		0.45	0.67	1.00		0.45
Intrusiveness	TFI 2	0.69	0.77	0.08	0.48	0.69	0.78	0.08	0.48
Intrusiveness	TFI 3	0.79	1.16	0.11	0.63	0.80	1.17	0.12	0.63
Sense of control	TFI 4	0.57	1.00		**0.33**	0.57	1.00		**0.33**
Sense of control	TFI 5	0.92	1.16	0.11	0.84	0.92	1.16	0.10	0.84
Sense of control	TFI 6	0.72	1.06	0.11	0.52	0.72	1.05	0.11	0.52
Cognitive	TFI 7	0.94	1.00		0.89	0.94	1.00		0.89
Cognitive	TFI 8	0.93	0.96	0.03	0.87	0.93	0.96	0.03	0.87
Cognitive	TFI 9	0.91	0.90	0.03	0.82	0.91	0.90	0.03	0.82
Sleep	TFI 10	0.88	1.00		0.78	0.88	1.00		0.78
Sleep	TFI 11	0.98	1.13	0.04	0.95	0.98	1.13	0.04	0.95
Sleep	TFI 12	0.91	1.04	0.04	0.82	0.91	1.04	0.04	0.82
Auditory	TFI 13	0.92	1.00		0.85	0.92	1.00		0.85
Auditory	TFI 14	0.98	1.10	0.03	0.97	0.98	1.10	0.03	0.97
Auditory	TFI 15	0.89	1.09	0.03	0.79	0.89	1.09	0.03	0.79
Relaxation	TFI 16	0.93	1.00		0.93	0.88	1.00		0.78
Relaxation	TFI 17	0.94	0.98	0.02	0.94	0.98	1.08	0.03	0.97
Relaxation	TFI 18	0.82	0.92	0.04	0.82	0.75	0.89	0.04	0.57
Quality of life	TFI 19	0.83	1.00		0.83	0.80	1.00		0.64
Quality of life	TFI 20	0.91	1.14	0.05	0.91	0.94	1.23	0.07	0.89
Quality of life	TFI 21	0.85	0.95	0.06	0.85	0.81	0.94	0.06	0.65
Quality of life	TFI 22	0.76	0.91	0.06	0.76	**0.43**	0.53	0.09	0.60
Cognitive	TFI 22	–	–	–	–	**0.40**	0.42	0.07	–
Emotional	TFI 23	0.89	1.00		0.89	0.89	1.00		0.80
Emotional	TFI 24	0.90	1.07	0.04	0.90	0.90	1.07	0.04	0.82
Emotional	TFI 25	0.83	0.87	0.04	0.83	0.83	0.87	0.04	0.68
**Second order factor**									
Functional impact of tinnitus	F1	0.80	1.48	0.14	0.62	0.78	1.47	0.14	0.62
	F2	0.92	1.71	0.17	0.83	0.91	1.71	0.16	0.83
	F3	0.87	2.38	0.10	0.75	0.87	2.37	0.10	0.75
	F4	0.62	1.83	0.15	**0.39**	0.62	1.84	0.15	**0.39**
	F5	**0.31**	0.79	0.15	**0.1**	0.30	0.77	0.16	**0.09**
	F6	0.83	2.36	0.12	0.69	0.84	2.26	0.12	0.70
	F7	0.87	2.10	0.13	0.75	0.86	2.01	0.14	0.74
	F8	0.91	2.28	0.12	0.83	0.92	2.29	0.12	0.84

**Table 5:** Reproducibility of Tinnitus Functional Index (TFI) scores: Intra-class correlations (ICC) and limits of agreement between two administrations. The TFI showed excellent stability over time as indicated by the high ICC values and acceptable test-retest agreement. Although most of the subscales were below 95% limits of agreement, it only suggested marginal measurement error. The smallest detectable change scores for the global TFI and subscales are comparable to the limits of agreement. ICC = Intra-class correlations; Mean diff = the mean difference scores between the repeated measure; SEM = Standard error of measurement; SDC = Smallest detectable change.

N = 44	Mean (±SD)		Reliability	Agreement				
			
Scale	Baseline	Retest	ICC (95%CI)	Mean diff	SEM	SDC	Limits of agreement	% of agreement
Tinnitus Functional index	45.3 (±20.1)	45.6(±19.4)	0.91 (0.84–0.95)	−0.3	8.1	22.4	22.2–22.7	93.2%
*Intrusiveness*	57.1 (±19.1)	58.8 (±21.3)	0.92 (0.82–0.96)	−1.7	7.6	21.1	19.4–22.7	93.2%
*Sense of control*	58.1 (±22.8)	57.6 (±20.9)	0.81 (0.65–0.90)	0.5	12.5	34.8	35.3–34.2	95.5%
*Cognitive*	39.2 (±38.2)	41.9 (±24.3)	0.89 (0.79–0.94)	−2.6	11.8	32.8	30.2–35.5	93.2%
*Sleep*	41.9 (±31.6)	41.2 (±30.1)	0.91 (0.83–0.95)	0.7	12.8	35.5	36.2–34.8	93.2%
*Auditory*	33.9 (±29.7)	36.1 (±30.2)	0.95 (0.90–0.97)	−2.3	9.6	26.6	24.3–28.9	93.2%
*Relaxation*	64.6 (±25.9)	62.9 (±25.3)	0.83 (0.69–0.91)	1.7	13.9	38.5	40.3–36.8	88.6%
*Quality of life*	35.1 (±26.1)	34.0 (±24.6)	0.86 (0.75–0.92)	1.1	12.6	34.9	36.0–33.8	93.2%
*Emotional*	36.0 (±28.1)	36.6 (±27.5)	0.87 (0.77–0.93)	−0.6	13.3	36.8	36.2–37.4	91.0%

**Table 6:** Correlations between global scores of all eight measures. The correlations between all eight measures indicate acceptable construct validity for the TFI. The strong correlations (>0.60) between the tinnitus questionnaires show high convergent validity, whilst the moderate correlations (>0.30) with the general health questionnaires show acceptable discriminant validity. TFI: Tinnitus Functional Index = THI; Tinnitus Handicap Inventory = THQ = Tinnitus Handicap Questionnaire, VAS-L = Visual analogue scale for loudness, PR-A = Percentage Rating Annoyance, BDI-II = Beck's Depression Inventory-II, BAI = Beck's Anxiety Inventory, WHOQOL-BREF = World Health Organisation Quality of Life-Bref.

	TFI	THI	THQ	VAS-L	PR-A	BDI	BAI	WHOQOL
TFI	1						
THI	0.82	1					
THQ	0.82	0.79	1				
VAS-L	0.46	0.41	0.29	1				
PR-A	0.58	0.58	0.41	0.42	1			
BDI	0.57	0.60	0.53	0.27	0.31	1		
BAI	0.39	0.43	0.43	0.20	0.19	0.67	1	
WHOQOL	−0.48	−0.52	−0.44	-0.16	−0.37	−0.55	−0.35	1

**Table 7:** Correlation coefficients (*r*), partial correlation coefficients (*pr*) and beta (β) values for the Tinnitus Functional Index (TFI) subscales and the Tinnitus Handicap Inventory (THI) global score, Tinnitus Handicap Questionnaire (THQ) global score, Beck's Depression Inventory-II (BDI-II), Beck's Anxiety Inventory (BAI); and World Health Organisation Quality of LifeeBREF (WHOQOL-BREF). *r* = Pearson's correlation coefficient; *Pr* = partial correlation coefficient; β = Standardised Beta values.

TFI subscale	*THI*			*THQ*			*BDI-II*			*BAI*			*WHOQOL*		
	*r*	*pr*	β	*r*	*pr*	β	*r*	*pr*	β	*r*	*pr*	β	*r*	*pr*	β
Intrusiveness	0.58	0.13	0.10	0.49	0.15	−0.11	0.29	−0.09	−0.10	0.14	−0.16	−0.19	−0.29	−0.00	−0.00
Sense of control	0.64	0.00	0.00	0.60	0.02	0.02	0.35	−0.19	−0.24	0.23	0.10	−0.14	−0.34	0.10	0.15
Cognition	0.72	0.09	0.09	0.73	0.19	0.17	0.58	0.25	0.34	0.39	0.14	0.22	−0.42	−0.01	−0.02
Sleep	0.58	0.19	0.14	0.54	0.21	0.15	0.40	0.07	0.07	0.28	0.09	0.10	−0.34	−0.03	−0.03
Auditory	0.22	0.06	−0.03	0.46	0.41	0.28	0.20	0.07	0.06	0.20	0.16	0.16	−0.04	0.13	0.12
Relaxation	0.66	0.10	0.09	0.63	0.10	0.09	0.44	0.06	0.07	0.27	−0.01	−0.02	−0.43	−0.12	−0.17
Quality of life	0.75	0.27	0.28	0.75	0.22	0.22	0.53	0.02	0.03	0.35	−0.02	−0.04	−0.47	−0.13	−0.20
Emotional	0.79	0.31	0.33	0.74	0.29	0.30	0.59	0.30	0.45	0.24	0.24	0.42	−0.53	−0.22	−0.36

**Table 8:** Correlation coefficients (*r*), partial correlation coefficients (*pr*) and beta (β) values for the Tinnitus Functional Index (TFI) subscales and the two major subscales of the Tinnitus Handicap Questionnaire (THQ). *r* = Pearson's correlation coefficient; *Pr* = partial correlation coefficient; β = Standardised Beta values.

	*THQ*	*factor*	1	*THQ*	*factor*	2
	*r*	pr	β	*r*	*pr*	β
Intrusiveness	0.48	–0.13	–0.09	0.27	–0.15	–0.12
Sense of control	0.65	0.04	0.04	0.25	–0.02	–0.02
Cognition	0.75	0.21	0.19	0.42	0.10	0.11
Sleep	0.64	0.31	0.21	0.16	–0.02	–0.02
Auditory	0.21	–0.01	–0.01	0.77	0.71	0.68
Relaxation	0.68	0.14	0.11	0.26	–0.03	–0.03
Quality of life	0.73	0.19	0.18	0.52	0.25	0.27
Emotional	0.81	0.36	0.37	0.31	0.01	0.23
