# Supplementary material for: Psychometric properties of the Tinnitus Functional Index (TFI): Assessment in a UK research volunteer population
Source: Hear Res. Author manuscript; Available in PMC 2017 Nov 30. (PMC5708524; doi:10.1016/j.heares.2015.09.009)
Supplement: Appendix [file NIHMS74986-supplement-Appendix.pdf]

Appendix

Appendix A

	Q1	Q2	Q3	Q4	Q5	Q6	Q7	Q8	Q9	Q10	Q11	Q12	Q13	Q14	Q15	Q16	Q17	Q18	Q19	Q20	Q21	Q22	Q23	Q24	Q25
Q1	1.00																								
Q2	0.55	1.00																							
Q3	0.56	0.48																							
Q4	0.26	0.32	0.35	1.00																					
Q5	0.38	0.53	0.60	0.53	1.00																				
Q6	0.50	0.59	0.51	0.41	0.66	1.00																			
Q7	0.39	0.45	0.49	0.42	0.69	0.50	1.00																		
Q8	0.34	0.40	0.48	0.43	0.66	0.46	**0.89**	1.00																	
Q9	0.35	0.46	0.48	0.48	0.70	0.50	**0.84**	**0.84**	1.00																
Q10	0.25	0.34	0.39	0.28	0.52	0.37	0.50	0.50	0.52	1.00															
Q11	0.27	0.34	0.40	0.25	0.50	0.37	0.50	0.49	0.50	0.86	1.00														
Q12	0.34	0.35	0.44	0.27	0.50	0.39	0.49	0.49	0.48	0.79	0.89	1.00													
Q13	0.25	0.26	0.21	0.13	0.18	0.22	0.26	0.30	0.26	0.09	0.07	0.10	1.00												
Q14	0.25	0.23	0.22	0.14	0.18	0.18	0.29	0.34	0.27	**0.11**	**0.11**	**0.14**	**0.90**	1.00											
Q15	0.25	0.25	0.18	0.09	0.17	0.18	0.28	0.30	0.25	0.06	0.07	**0.11**	0.82	0.87	1.00										
Q16	0.34	0.46	0.48	0.44	0.64	0.58	0.61	0.58	0.62	0.52	0.50	0.51	0.23	0.21	0.19	1.00									
Q17	0.35	0.47	0.53	0.47	0.67	0.60	0.69	0.65	0.71	0.59	0.59	0.60	0.22	0.22	0.20	0.87	1.00								
Q18	0.26	0.45	0.41	0.43	0.57	0.53	0.50	0.46	0.52	0.44	0.42	0.43	0.21	0.19	0.17	0.81	0.74	1.00							
Q19	0.34	0.33	0.47	0.25	0.52	0.40	0.54	0.56	0.55	0.31	0.32	0.34	0.39	0.39	0.35	0.50	0.56	0.37	1.00						
Q20	0.31	0.42	0.56	0.39	0.66	0.46	0.64	0.66	0.67	0.38	0.40	0.40	0.30	0.30	0.28	0.60	0.67	0.51	0.76	1.00					
Q21	0.31	0.28	0.51	0.28	0.53	0.37	0.59	0.62	0.58	0.36	0.36	0.39	0.37	0.39	0.33	0.45	0.55	0.37	0.78	0.75	1.00				
Q22	0.29	0.35	0.43	0.34	0.58	0.38	0.67	0.68	0.66	0.38	0.42	0.41	0.27	0.32	0.31	0.47	0.53	0.41	0.59	0.68	0.64	1.00			
Q23	0.29	0.43	0.52	0.39	0.68	0.51	0.65	0.63	0.65	0.44	0.46	0.49	0.14	0.17	0.13	0.56	0.65	0.48	0.57	0.73	0.66	0.67	1.00		
Q24	0.34	0.49	0.54	0.45	0.70	0.60	0.66	0.62	0.71	0.46	0.46	0.50	0.16	0.16	0.17	0.58	0.67	0.51	0.56	0.72	0.60	0.60	**0.81**	1.00	
Q25	0.24	0.39	0.48	0.39	0.65	0.46	0.61	0.60	0.66	0.43	0.41	0.42	0.20	0.20	0.19	0.50	0.60	0.45	0.51	0.69	0.61	0.55	0.74	0.74	1

Values presented in bold are below or above the recommended criteria (<0.30 to >0.85).

Appendix B

	Scale items	Percentage of responses for items on the TFI											Mean (±SD)	
		0	1	2	3	4	5	6	7	8	9	10		
Int1	What percentage of your time awake were you consciously aware of your tinnitus?	0.4	6.4	7.1	8.1	7.1	10.2	8.1	11.0	17.3	12.4	12	6.20	(2.79)
Int2	How strong or loud was your tinnitus?	0.0	1.4	3.2	9.5	10.2	12.4	12.0	20.8	19.8	7.4	3	6.14	(2.10)
Int3	What percentage of your time awake were you annoyed by your tinnitus?	9.2	23.3	13.1	12.0	4.2	11.7	5.7	9.5	6.7	3.2	1	3.58	(2.76)
SOC4	Did you feel in control in regard to your tinnitus?	5.3	5.3	8.1	11.0	6.7	9.9	5.3	7.4	10.2	9.2	**22**	5.95	(3.27)
SOC5	How easy was it for you to cope with your tinnitus?	6.7	8.8	10.6	14.5	10.2	17.0	9.9	15.5	5.3	0.7	1	4.23	(2.38)
SOC6	How easy was it for you to ignore with your tinnitus?	2.1	5.3	6.7	10.2	6.4	13.4	10.2	12.4	14.1	9.9	9	5.84	(2.74)
Cog7	How much did your tinnitus interfere with your ability to concentrate?	**16.3**	11.7	14.1	8.1	5.3	9.5	11.0	12.0	7.1	3.2	2	3.86	(2.91)
Cog8	How much did your tinnitus interfere with your ability to think clearly?	**22.6**	12.4	10.2	11.0	7.1	11.3	7.8	7.8	7.1	1.8	1	3.35	(2.81)
Cog9	How much did your tinnitus interfere with your ability to focus attention on other things beside your tinnitus?	**19.1**	14.8	11.3	13.4	7.4	8.5	8.1	7.8	7.1	2.1	0	3.32	(2.73)
Slp10	How often did your tinnitus make it difficult to fall asleep or stay asleep?	**19.1**	9.9	12.0	7.8	8.1	5.3	4.9	10.6	10.2	4.2	8	4.18	(3.35)
Slp11	How often did your tinnitus cause you difficulty in getting as much sleep as you needed?	**23.0**	11.3	10.6	9.2	4.2	5.7	7.4	7.8	7.8	5.3	8	3.92	(3.42)
Slp12	How much of the time did your tinnitus keep you from sleeping as deeply or as peacefully as you would have liked?	**23.3**	10.6	11.7	10.6	3.9	7.8	3.9	6.4	8.1	7.4	6	3.83	(3.39)
Aud13	How much did your tinnitus interfere with your ability to hear clearly?	**18.4**	13.4	10.2	11.3	7.8	13.1	7.8	8.1	7.1	1.4	1	3.51	(2.76)
Aud14	How much did your tinnitus interfere with your ability to understand people who are talking?	**25.4**	11.0	11.3	11.7	8.1	7.8	7.4	6.4	8.5	1.1	1	3.20	(2.85)
Aud15	How much did your tinnitus interfere with your ability to follow conversations in a group or at meetings?	**23.3**	9.9	10.2	10.6	10.2	6.0	6.4	9.2	5.3	5.3	4	3.61	(3.10)
Relx16	How much did your tinnitus interfere with your quiet resting activities?	8.8	7.4	8.8	8.1	7.1	9.2	9.5	12.4	14.8	6.7	7	5.17	(3.06)
Relx17	How much did your tinnitus interfere with your ability to relax?	9.5	11.0	9.9	8.1	9.9	11.0	6.4	14.5	8.8	7.1	4	4.62	(2.98)
Relx18	How much did your tinnitus interfere with your ability to peace and quiet?	4.9	4.2	5.3	7.4	6.0	6.0	5.3	10.6	12.0	12.7	**25**	6.62	(3.18)
QOL19	How much did your tinnitus interfere with your enjoyment of social activities?	**32.9**	11.0	12.4	9.5	4.9	7.4	5.3	7.4	5.3	1.8	2	2.84	(2.92)
QOL20	How much did your tinnitus interfere with your enjoyment of life?	**25.8**	13.1	11.7	11.3	6.0	6.4	6.4	7.4	4.6	3.9	4	3.23	(3.05)
QOL21	How much did your tinnitus interfere with your relationships with family, friends and other people?	**37.5**	17.3	9.5	7.4	4.2	8.8	4.6	3.5	3.5	2.5	1	2.33	(2.72)
QOL22	How often did your tinnitus cause you to have difficulty performing your work or other tasks, such as home maintenance, school work, or caring for children or others?	**32.2**	15.5	11.7	8.1	4.2	7.4	3.5	8.1	5.3	2.8	1	2.74	(2.90)
Emo23	How anxious or worried has your tinnitus made you feel?	**23.7**	16.6	14.8	10.6	3.5	8.5	5.7	8.1	4.9	1.8	2	2.99	(2.81)
Emo24	How bothered or upset have you been because of your tinnitus?	14.8	15.9	14.8	7.4	6.7	12.0	5.7	8.8	5.3	6.0	2	3.72	(2.97)
Emo25	How depressed were you because of your tinnitus?	**41.0**	14.1	10.2	8.1	3.9	7.8	5.7	3.9	2.5	2.5	0	2.20	(2.63)

Values presented in bold exceed the recommended criteria (endorsed by >15% of respondents).
